# Vaseline gauze packing for the treatment of acute hemorrhagic rectal ulcer: Two case reports

**DOI:** 10.1097/MD.0000000000033411

**Published:** 2023-03-31

**Authors:** Sung Kyun Yim, Chang Hun Lee, Seong-Hun Kim, Sang Wook Kim, Seung Young Seo

**Affiliations:** a Department of Internal Medicine, Research Institute of Clinical Medicine of Jeonbuk National University-Biomedical Research Institute of Jeonbuk National University Hospital, Jeonju, Republic of Korea.

**Keywords:** acute hemorrhagic rectal ulcer, lowers gastrointestinal bleeding, rectal ulcer, Vaseline gauze

## Abstract

Acute hemorrhagic rectal ulcer (AHRU) is a relatively rare condition characterized by sudden onset, painlessness, and massive hematochezia in patients with severe underlying conditions. When AHRU is encountered, they can often be successfully controlled endoscopically, though recurrent bleeding is common and an alternative treatment must be sought if initial endoscopic treatment fails. We report 2 cases of AHRU which were successfully treated with Vaseline gauze packing after the failure of endoscopic hemostasis.

**Patient concerns:**

The first patient was an 88-year-old female that visited our emergency department with hematochezia. She was immobilized because of a left pelvic bone fracture resulting from a slip-down. The initial endoscopy showed fresh blood in her rectum with diffuse ulceration near the dentate line but no active bleeding. However, Massive hematochezia has recurred during conservation. A second patient, an 86-year-old female, debilitated because of schizophrenia, dementia, and past subdural hemorrhage, visited our emergency department, also with massive hematochezia. Her initial endoscopy showed deep ulceration near the dentate line. After admission, she experienced massive hematochezia from an AHRU with an exposed vessel but endoscopic hemostasis failed to control bleeding.

**Diagnoses:**

Both patients were diagnosed as AHRU based on the endoscopic findings.

**Interventions:**

In both cases, Vaseline gauze packing was performed for bleeding control.

**Outcomes:**

After Vaseline gauze packing, no further bleeding occurred and follow-up endoscopy showed definitive improvement of ulcers.

**Lessons:**

Based on these cases, we suggest that Vaseline gauze packing may be the alternative treatment for the AHRU which is located near the dentate line when endoscopic hemostasis is difficult or failed. Although further research is needed, Vaseline gauze packing has several potential advantages for the treatment of AHRU, especially in cases involving critically ill elderly patients.

## 1. Introduction

Solitary rectal ulcer syndrome is an uncommon disease that may present with rectal bleeding, straining during defecation, and a sense of incomplete evacuation.^[1]^ Occasionally, bleeding from rectal ulcers can be life-threatening. This type of acute critical bleeding from rectal ulcer was named acute hemorrhagic rectal ulcer (AHRU) by Soeno et al in 1981.^[2]^ AHRUs are characterized by their sudden onset, painlessness, and massive hematochezia, in patients with severe underlying conditions (e.g. cerebral vascular disease, diabetes mellitus, and cardiovascular disease).^[3]^ One recent report showed that AHRU is an important cause of death in intensive care unit settings.^[4]^ In many cases, AHRU can be managed endoscopically using endoscopic clips, an epinephrine injection, hemostatic forceps, or argon plasma coagulation.^[5]^ However, in some cases, endoscopic management may prove difficult, particularly when the bleeding site is located at the distal rectum near the dentate line. In these instances, alternative treatments must be provided.

Vaseline gauze is a widely used treatment for cutaneous wounds. Vaseline gauze retains moisture and prevents synechiae formation, thereby promoting wound healing.^[6]^ Vaseline gauze packing can be used for nasal bleeding and after nasal surgery,^[7]^ and it can serve as a tamponade in other cases of postoperative bleeding after hemorrhoidectomy.^[8]^ Considering its wound healing and tamponade effect, Vaseline gauze packing may be used for bleeding control in cases of AHRU in the distal rectum. We report 2 cases in which AHRU bleeding in the distal rectum was managed with Vaseline gauze packing; in both cases, the treatment successfully controlled the bleeding and the ulcer showed accelerated healing. This case report was approved by the Institutional Review Board of Jeonbuk National University Hospital (IRB No. 2022-12-055) and written informed consent was obtained from the patient and legal guardian/next of kin for publication.

## 2. Case report

### Case 1:

**Figure 1.**
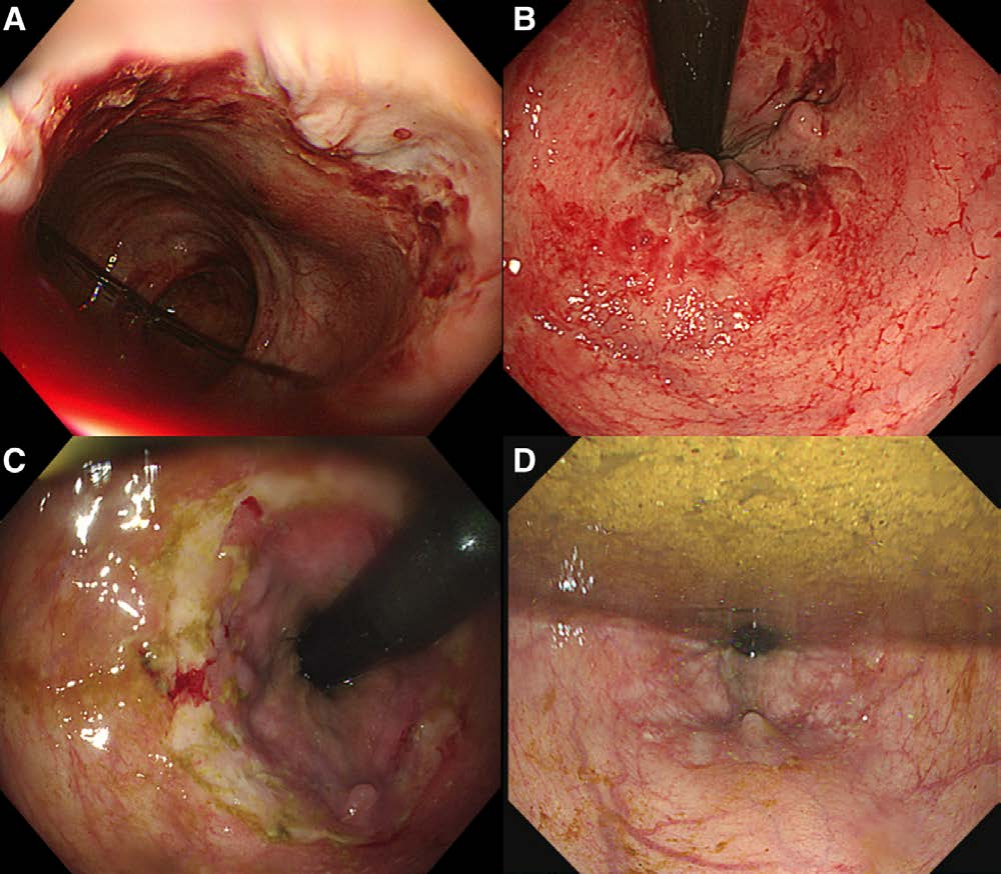
Sigmoidoscope results from case report 1. A large amount of fresh blood was noted in (A) the rectum with diffuse ulcerations near (B) the dentate line. However, there was no further active bleeding and endoscopic hemostasis was not performed. (C) On follow-up on hospital day (HD) 3 more definite ulcerations near the dentate line and possible bleeding spots were noted. Because of recurred massive bleeding on HD 5 Vaseline gauze packing with Vaseline gauze roll was done and removed on HD 9. (D) Follow-up sigmoidoscopy on HD 11 showed marked improvement of the rectal ulcer. HD = hospital day.

An 88-year-old female visited our emergency department with hematochezia. She was immobilized because of left pelvic bone fracture after a slip-down that had occurred a week prior, for which she was receiving conservative treatment in a local orthopedic hospital. The patient had underlying diabetes mellitus and hypertension and was constipated after immobilization. On arrival, the patient’s blood pressure was 177/88 mm Hg, heart rate was 97 bpm, and body temperature was 36.3°C. Laboratory examination revealed the following: leukocytes = 12210/μL (neutrophils = 95.0%), hemoglobin = 12.5 g/dL, platelets = 286 × 103/μL, blood urea nitrogen = 19 mg/dL, and creatinine = 0.77 mg/dL. A sigmoidoscopy revealed fresh blood in her rectum and diffuse ulcerations near the dentate line (Fig. 1A and B). In the absence of active bleeding, endoscopic hemostasis was not performed and the patient was admitted to the orthopedics department for management of the pelvic bone fracture. The follow-up sigmoidoscopy on hospital day (HD) 3 showed more definite ulcerations near the dentate line and possible bleeding spots (Fig. 1C). With no definitive signs of bleeding, the orthopedics department considered surgical management for the pelvic bone fracture. However, massive hematochezia was noted on HD 5. As the AHRU was diffuse and located near the dentate line, we performed Vaseline gauze packing in lieu of endoscopic hemostasis. A roll of Vaseline gauze was completely packed into the rectum manually. After this procedure, the patient showed no further signs of bleeding, and the Vaseline gauze was removed on HD 9. A follow-up sigmoidoscopy performed on HD 12 showed resolution of the previous rectal ulcerations (Fig. 1D). In light of the poor general condition of the patient, her family decided not to undergo surgery for the pelvic bone fracture, and was subsequently discharged to the local hospital for conservative treatment.

### Case 2:

**Figure 2.**
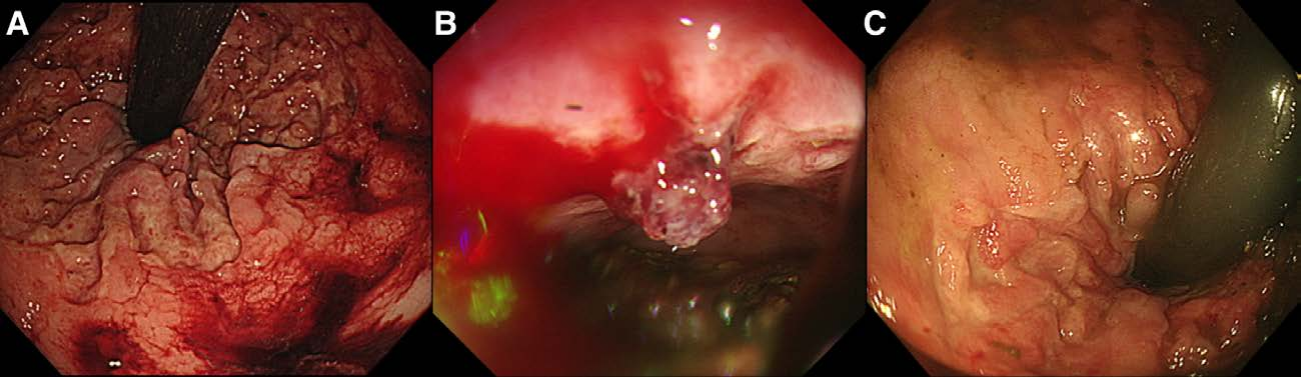
Sigmoidoscope results from case report 2. (A) Initial sigmoidoscopy showed blood in the rectum with a large circumferential ulcer near the dentate line, but no active bleeding or exposed vessel was suspected. (B) Massive bleeding was noted on hospital day (HD) 6 and the follow-up sigmoidoscopy showed an exposed vessel with active bleeding. Endoscopic hemostasis with epinephrine injection and clipping failed, after which Vaseline gauze packing was performed. (C) The gauze was removed on HD 11 and the follow-up endoscopy on HD 12 showed no exposed vessel and regeneration of the previous ulcer base. HD = hospital day.

An 86-year-old female visited our emergency department with massive hematochezia. She was debilitated and had been previously diagnosed with schizophrenia, dementia, and a past subdural hemorrhage. On arrival the patient’s blood pressure was 107/72 mm Hg, heart rate was 110 bpm, and body temperature was 37.2°C. Laboratory examination revealed the following: leukocytes = 9370/μL, hemoglobin = 7.5 g/dL, platelets = 181 × 103/μL, blood urea nitrogen = 49 mg/dL, and creatinine = 1.21 mg/dL. Sigmoidoscopy revealed fresh blood in her rectum and diffuse deep ulcerations near the dentate line, but no further active bleeding or exposed vessels were noted (Fig. 2A). The patient was admitted and supportive management was provided. As there was no further bleeding, an oral diet was resumed on HD 4. However, the patient had a massive hematochezia on HD 6 and sigmoidoscopy revealed a large exposed vessel with active bleeding at the dentate line (Fig. 2B). Endoscopic hemostasis with epinephrine injection and clipping was attempted, but the poor location and large size of the vessel mitigated the effects of the procedure, and bleeding persisted. We performed Vaseline gauze packing in lieu of reattempting endoscopic hemostasis, after which the bleeding stopped. The gauze was removed on HD 11 and a follow-up endoscopy on HD12 showed no exposed vessel and regeneration of the previous ulcer base (Fig. 2C).

## 3. Discussion

AHRU often occurs in elderly or critically ill patients with severe comorbidities,^[3,4]^ and in many cases, it can be a life-threatening event with a poor outcome. In the reported literature, as many as 60% of patients with AHRU have died, although in most cases the cause of death has been attributed to comorbidities and not the bleeding itself.^[3–5,9]^ Patients with AHRU usually require transfusions, highlighting the importance of prompt diagnosis and management.^[2–4]^ Endoscopic treatment is often selected as the initial treatment modality.^[5]^ Endoscopic hemostasis can be performed using coagulation forceps, argon plasma coagulation, endoscopic clips, a heat probe, or epinephrine-saline injection therapy, and has a success rate of 84 to 97.2%.^[4,5]^ However, recurrent bleeding is common after the endoscopic hemostasis (20–48.3%), and additional endoscopic hemostasis procedures are often needed.^[4,5]^ While endoscopic hemostasis may control the bleeding, it does not address the ulcer itself, and the high vascularity of the recto-anal area may be responsible for AHRU’s high recurrence rate. In our cases, recurrent bleeding from AHRU was noted and endoscopic hemostasis was difficult or failed. Vaseline gauze packing was done as an alternative treatment for AHRU and revealed marked improvement in ulceration within only 6 to 7 days. Thus, Vaseline gauze packing may have accelerated the healing process of the ulcer. The mechanism of the healing effect for the AHRU in Vaseline gauze packing is uncertain. However, several possible mechanisms can be considered. In the healing process of the ulcer, bacterial colonization may delay the healing process.^[10]^ Bacteria are known to mediate inflammatory cytokines and reactive oxygen species by their cell wall products and thus causing inflammation and tissue damage.^[11]^ Although the evidence is inconclusive, Vaseline may have an effect of robust modulation of antimicrobials and epidermal differentiation barrier measures.^[12]^ Another role of Vaseline in wound healing is its moisture-retention ability and prevention of synechiae formation.^[6]^ In short, it may have been the placement of Vaseline on the rectal ulcers that accelerated their healing. In addition, Vaseline gauze packing has a tamponade effect for AHRU bleeding.

The AHRU are commonly located 1 to 7 cm from the dentate line and occasionally at the dentate line.^[3,5,9]^ Due to the limited space and the close distance to the anus, endoscopic hemostasis can be challenging when the bleeding site is close to the dentate line. When endoscopic treatment is not available or failed to achieve sufficient hemostasis, an alternative treatment modality is needed. Surgical hemostasis with trans-anal suture ligation is a commonly used option.^[4,5,9]^ Trans-anal suture ligation often successfully controls the bleeding, but limited orifice and identification of the bleeding vessel may be difficult and result in insufficient hemostasis.^[3]^ One other treatment option is trans-arterial embolization;^[9]^ however, patients with AHRU are generally elderly and diagnosed with severe underlying conditions such as renal failure. One previous account of AHRU patients in an intensive care unit reported that 55% had renal failure.^[4]^ Thus, in concern of contrast-induced nephropathy, trans-arterial embolization can be risky in AHRU. Moreover, because the rectal blood supply originates from the bilateral internal iliac arteries and the inferior mesenteric artery, identification of the bleeding focus can take longer and require greater effort than when the bleeding occurs in other sites of the gastrointestinal tract.^[13]^ Compounding the difficulty, the rectum has a high vascular supply and multiple collateral channels, and the inferior mesenteric artery can be hard to access in elderly patients.

The application of a gauze tamponade is another means of controlling AHRU.^[9]^ Gauze tamponades are commonly used by clinics in response to anal or nasal bleeding. One previous report described a very high rebleeding rate and frequent need for trans-anal ligation (8 out of 11 AHRU patients) after the application of a simple gauze tamponade, though the type of gauze used was not described.^[9]^ It is true that not all the patients with AHRU managed with Vaseline gauze packing experience good outcomes as in our cases. A search of the medical records of AHRU patients treated at our institution between January 2020 and March 2022 identified 6 cases in addition to those herein reported. Three of these cases were successfully treated with endoscopic clips. One case was initially controlled by epinephrine injection, but the patient experienced recurrent bleeding and underwent surgical ligation. One patient’s AHRU was initially managed with clipping, but Vaseline gauze packing was done after rebleeding occurred. In this case, bleeding recurred after the removal of the gauze and surgical ligation was performed. One patient was initially managed with Vaseline gauze packing but underwent surgical ligation when bleeding recurred. In total, 4 patients (including our 2 cases reported herein) underwent Vaseline gauze packing, with 2 of these 4 subsequently requiring additional treatment. We note, however, that the AHRU which required further treatment in addition to Vaseline gauze packing was located in a more oral side of the rectum (2 cm above the dentate line) than in those whose condition was successfully managed by Vaseline gauze packing treatment. In light of this observation, we suggest that hemostasis with Vaseline gauze packing for AHRU is least likely to be achieved, especially when AHRU is located >2 cm above the dentate line.

## 4. Conclusion

Based on our review of these cases, we suggest that Vaseline gauze packing can be employed as a treatment when AHRU is located within 2 cm of the dentate line and when endoscopic treatment is not available or failed. Vaseline gauze packing may have a hemostatic effect on AHRU by its application of compression pressure, and the presence of the gauze may accelerate ulcer healing. Although not all cases of AHRU will resolve after Vaseline gauze packing, the procedure has been shown to frequently control active bleeding. The minimal invasiveness of this procedure suggests that it should be considered a safe and relatively easy-to-perform treatment for elderly and critically ill patients with severe comorbidities with AHRU located near the dentate line. Our case report suggests that additional research is warranted to confirm the beneficial effect of this treatment on ulcer healing and identify the best candidate recipients.

## Author contributions

**Conceptualization:** Sang Wook Kim, Seung Young Seo.

**Data curation:** Sung Kyun Yim, Seong-Hun Kim.

**Resources:** Chang Hun Lee, Seong-Hun Kim.

**Validation:** Sang Wook Kim, Seung Young Seo.

**Visualization:** Sung Kyun Yim, Chang Hun Lee.

**Writing – original draft:** Sung Kyun Yim.

**Writing – review & editing:** Sung Kyun Yim, Sang Wook Kim, Seung Young Seo.
